# Vancomycin population pharmacokinetics during extracorporeal membrane oxygenation therapy: a matched cohort study

**DOI:** 10.1186/s13054-014-0632-8

**Published:** 2014-11-22

**Authors:** Katia Donadello, Jason A Roberts, Stefano Cristallini, Marjorie Beumier, Kiran Shekar, Frédérique Jacobs, Asmae Belhaj, Jean-Louis Vincent, Daniel de Backer, Fabio Silvio Taccone

**Affiliations:** Department of Intensive Care, Erasme Hospital, Université Libre de Bruxelles, Route de Lennik, 808 – 1070 Brussels, Belgium; Burns Trauma and Critical Care Research Centre, The University of Queensland, Brisbane, Australia; Critical Care Research Group, Adult Intensive Care Services, The Prince Charles Hospital, Brisbane, Australia; Department of Infectious Diseases, Erasme Hospital, Université Libre de Bruxelles, Route de Lennik, 808 – 1070 Brussels, Belgium; Department of Thoracic Surgery, Erasme Hospital, Université Libre de Bruxelles, Route de Lennik, 808 – 1070 Brussels, Belgium

## Abstract

**Introduction:**

The aim of this study was to describe the population pharmacokinetics of vancomycin in critically ill patients treated with and without extracorporeal membrane oxygenation (ECMO).

**Methods:**

We retrospectively reviewed data from critically ill patients treated with ECMO and matched controls who received a continuous infusion of vancomycin (35 mg/kg loading dose over 4 hours followed by a daily infusion adapted to creatinine clearance, CrCl)). The pharmacokinetics of vancomycin were described using non-linear mixed effects modeling.

**Results:**

We compared 11 patients treated with ECMO with 11 well-matched controls. Drug dosing was similar between groups. The median interquartile range (IQR) vancomycin concentrations in ECMO and non-ECMO patients were 51 (28 to 71) versus 45 (37 to 71) mg/L at 4 hours; 23 (16 to 38) versus 29 (21 to 35) mg/L at 12 hours; 20 (12 to 36) versus 23 (17–28) mg/L at 24 hours (ANOVA, *P* =0.53). Median (ranges) volume of distribution (Vd) was 99.3 (49.1 to 212.3) and 92.3 (22.4 to 149.4) L in ECMO and non-ECMO patients, respectively, and clearance 2.4 (1.7 to 4.9) versus 2.3 (1.8 to 3.6) L/h (not significant). Insufficient drug concentrations (that is drug levels <20 mg/dL) were more common in the ECMO group. The pharmacokinetic model (non-linear mixed effects modeling) was prospectively validated in five additional ECMO-treated patients over a 6-month period. Linear regression analysis comparing the observed concentrations and those predicted using the model showed good correlation (r^2^ of 0.67; *P* <0.001).

**Conclusions:**

Vancomycin concentrations were similar between ECMO and non-ECMO patients in the early phase of therapy. ECMO treatment was not associated with significant changes in Vd and drug clearance compared with the control patients.

## Introduction

Extracorporeal membrane oxygenation (ECMO) is a temporary life support system, which is increasingly used for the management of acute severe cardiac and/or respiratory failure [1]. Veno-venous (VV) ECMO is used to treat severe respiratory failure, and veno-arterial (VA) ECMO can provide cardiovascular and respiratory support for patients with severe circulatory shock and heart failure [2]. Antibiotics are commonly required during ECMO therapy in patients who are infected, so that it is essential to understand any potential changes in antibiotic pharmacokinetics (PK) that may occur during ECMO to enable rational dose adjustments to be made [3]. There are currently relatively few data available regarding antibiotic PK during ECMO and patients are generally managed with similar antibiotic dosing regimens to those used in patients who are not receiving ECMO. This approach may, however, be flawed because ECMO and sepsis have been shown to result in altered antibiotic PK, leading to sub-therapeutic drug concentrations [4-6]. Moreover, ECMO equipment can introduce additional confounding factors, from the circuit itself (with associated drug sequestration) and the associated systemic inflammation (with vasodilation and capillary leak) [3].

In a recent review, Shekar *et al*. highlighted that the major PK changes commonly associated with ECMO are an increased volume of distribution (Vd) and decreased drug clearance (CL) [7], although the extent of such changes remains poorly characterized, especially in adult patients. Moreover, emerging *in vitro/ex vivo* data on dose requirements for adult patients on ECMO suggest that standard drug regimens may be inadequate [8], because of significant drug sequestration on the ECMO tubing and/or membrane leading to lower plasma concentrations [9].

Vancomycin remains one of the first options for treating nosocomial infections caused by methicillin-resistant *Staphylococcus aureus* (MRSA) or other resistant Gram-positive bacteria, such as coagulase-negative staphylococci and ampicillin-resistant enterococci [10]. In the critical care setting, continuous infusion (CI) of vancomycin may enable a more rapid and consistent attainment of target drug concentrations than standard intermittent administration [11]. As vancomycin is expected to be poorly soluble in organic materials, drug concentrations and CL would be only minimally affected by ECMO, while the use of priming fluids and the cardiovascular alterations, which are often associated with the use of ECMO, would contribute to increase its Vd [9]. Furthermore, vancomycin can be nephrotoxic and patients undergoing ECMO treatment are at high risk to develop renal failure because pre-existing kidney damage is frequent in this setting [12]. Thus, monitoring of vancomycin levels is fundamental in such patients, especially in the case of prolonged therapy [13]. Moreover, when renal replacement therapy (RRT) is initiated in those patients, the risk of insufficient drug levels when standard regimens are used is around 20% and also warrants close monitoring of vancomycin concentrations [14].

Only one study has previously evaluated vancomycin concentrations and PK in adult patients undergoing ECMO [15]. This study evaluated vancomycin administration as an intermittent infusion with data compared to neonates or pediatric data on ECMO, but not to a critically ill adult population without ECMO. Thus, the aim of this study was, therefore, to compare the population PK of vancomycin given as CI in critically ill patients treated with and without ECMO. Our hypothesis is that the use of ECMO would result in an increased Vd and unchanged CL of vancomycin when compared to critically ill patients not treated with ECMO.

## Materials and methods

### Extracorporeal membrane oxygenation patients and data collection

We reviewed the medical charts of all adult (>18 years old) patients who received ECMO support (VV, VA, or both) and, at the same time, were given a continuous infusion of vancomycin, either as monotherapy or combined with other antibiotics, in our multidisciplinary 35-bed Department of Intensive Care (Brussels, Belgium) between January 2011 and May 2012. Continuous infusion of vancomycin is the standard of care in our ICU. Patients were identified using the department’s patient data monitoring system (PDMS) (Picis Inc., Wakefield, MA, USA).

We included all adult patients who had serial measurements of serum vancomycin concentrations during the first 24 h of treatment during ECMO. Patients who had previously received vancomycin by intermittent infusion (within 48 h of the start of the CI) were excluded, as were those where vancomycin and ECMO treatment were not simultaneous, and those with pregnancy, burns or cystic fibrosis. No patient included in previous publications [16-18] was included in the present study. The protocol was approved by the Ethics Committee of Erasme Hospital, which waived the need for informed consent because of the retrospective nature of the study.

The following data were collected for all patients: demographics; pre-existing chronic diseases; admission diagnosis; ECMO indications and settings; fluid balance; and microbiological findings. The severity of illness of each patient was assessed using the acute physiology and chronic health evaluation (APACHE) II score [19] at ICU admission and the sequential organ failure assessment (SOFA) score [20] at initiation of antibiotic therapy. Use of vasopressor agents or mechanical ventilation was recorded, as was the length of ICU stay and outcome. Creatinine clearance (CrCl) was calculated from the 24-h urine collection, using the following formula:

$$ \begin{array}{l}\mathrm{CrCl},\ \mathrm{mL}/\mathrm{minute} = \left(\left(\mathrm{Urine}\ \mathrm{output},\ \mathrm{mL}\right)*\left(\mathrm{Urinary}\ \mathrm{creatinine},\ \mathrm{mg}/\mathrm{dL}\right)\right)\\ {}/\left(\left(\mathrm{Serum}\ \mathrm{creatinine},\ \mathrm{mg}/\mathrm{dL}\right)*\left(\mathrm{Time}\ \mathrm{of}\ \mathrm{urine}\ \mathrm{collection},\ \mathrm{minutes}\right)\right).\end{array} $$

### Continuous renal replacement therapy

The decision to initiate continuous renal replacement therapy (CRRT) was made according to standard practice [17]; CRRT was performed using a double-lumen catheter inserted into a central vein. Continuous veno-venous hemodiafiltration (CVVHDF) or hemofiltration (CVVHF) were performed using a Prisma-Flex machine (Gambro Hospal, Bologna, Italy), with polyacrilonitrile (AN69 - Hospal, Meyzieu, France) or polysulfone (PS, Gambro Lundia AB, Lund, Sweden) hemofilters. Anticoagulation was obtained using a continuous infusion of either heparin or citrate. Initial CRRT settings were as follows: blood flow 130 to 150 mL/minute; ultrafiltration rate 15 to 20 mL/kg/h; dialysate rate 15 to 20 mL/kg/h. Fluid removal was decided according to patient’s condition. CRRT intensity was calculated as:

$$ \left(\mathrm{Dialysate}\ \mathrm{rate}\ \left(\mathrm{mL}/\mathrm{h}\right) + \mathrm{Ultrafiltrate}\ \mathrm{rate}\ \left(\mathrm{mL}/\mathrm{h}\right)\right)/\mathrm{Weight}\ \left(\mathrm{kg}\right). $$

### Extracorporeal membrane oxygenation circuit and management

All ECMO equipment was implanted surgically with peripheral (femoro-femoral if VA and femoro-jugular if VV) or central heparin-coated cannulation (20- to 22-Fr arterial cannula and 22- 24-Fr venous cannula, Edwards Lifesciences, Irvine, CA, USA). A centrifugal blood pump (Revolution blood pump, Sorin, Milan, Italy) was initially set at a blood flow of 3 to 4 L/minute. The ECMO circuit was primed with 700 mL of a balanced crystalloid infusion (Plasmalyte, Baxter Healthcare Corporation, Deerfield, IL, USA). In patients with peripheral VA implantation, an anterograde single-lumen 8-Fr catheter (Arrow Inc, Reading, PA, USA) was placed to prevent limb ischemia. A heat exchanger (Blanketrol II, Sub-Zero Products Inc., Cincinnati, OH, USA) was used to maintain body temperature at 37°C.

### Vancomycin treatment and measurements

Vancomycin (Vancocin®; Eli Lilly, Saint-Cloud, France) was reconstituted according to the manufacturer’s guidelines. The drug was given as a 35 mg/kg loading dose over 4 h followed by a CI dose adapted to CrCl to provide serum concentrations of 20 to 30 mg/L (considered appropriate) (Table 1); this drug regimen has been the standard of care in our institution since 2011 and was adapted according to a previous publication [18]. Doses were not changed during the first 24 h of therapy; afterwards, the daily drug regimen was adapted using a specific approach, as previously published [16-18]: if the serum vancomycin concentration was <20 μg/mL (considered insufficient), an additional dose of 500 to 1,000 mg was given followed by an increase in the daily dose by 500 to 1,000 mg. If the concentration was >30 μg/mL (considered excessive), the CI was discontinued for 4 to 8 h and the daily dose reduced by 500 to 1000 mg per day.

**Table 1:** **Daily vancomycin doses according to the creatinine clearance (CrCL)**

	**Daily dose**
**CrCL, L/minute**	
>150	45 mg/kg
120 to 150	40 mg/kg
80 to 120	35 mg/kg
50 to 80	25 mg/kg
25 to 50	14 mg/kg
<25 or oliguria	7 mg/kg
**Continuous renal replacement therapy**	14 mg/kg

Oliguria was defined as urine output ≤0.5 mL/kg/h.

Blood samples (3 mL) for measurement of drug concentrations were retrieved at 4 (T1), 12 (T2) and 24 h (T3) after the start of therapy as part of standard care and were immediately sent to the central laboratory. The nursing staff recorded the exact sampling time in the PDMS system. Serum concentrations of vancomycin were determined by particle-enhanced turbidimetric inhibition immunoassay (Dimension® XPand®; Siemens Healthcare Diagnostics, Newark, DE, USA). The limit of quantification and the total imprecision of the assay were 0.8 mg/L and <5%, respectively.

### Matched controls

Using an institutional database of all ICU patients without ECMO who received the same vancomycin regimen (n = 107) during the same period, ICU ECMO patients were matched (1:1) with non-ECMO ICU patients according to four criteria: 1) renal function (either same CrCl, with a range of eligibility for matching of ±10 mL/minute, or if on CRRT, the same CRRT intensity, with a range of eligibility for matching of ±5 mL/kg/minute); 2) estimated total body weight; 3) SOFA score at the time of treatment initiation; and 4) age (range of eligibility for matching of ±5 years). The use of such variables for the matching process was decided based on the impact of estimated body weight and renal function on drug Vd and CL, respectively, as well as the importance of the disease severity, multiple organ dysfunction and age on drug PK and metabolism [4,5,7].

### PK data

The concentration-time data for serum vancomycin concentrations were described using non-linear mixed-effects modeling (NONMEM version 7.2.0, ICON Development Solutions, Ellicott City, MD, USA) [21]. A Digital Fortran compiler was used and the runs were executed using Wings for NONMEM [22]. Data were analyzed using the first-order conditional estimation method with interaction. One- and two-compartment linear models were both evaluated. Between-subject variability was calculated using an exponential variability model. Residual unexplained variability was evaluated as additive, exponential or combined (additive plus exponential). Visual inspection of diagnostic scatter plots and the NONMEM objective function value (OFV) were used to evaluate goodness of fit. Statistical comparison of nested models was undertaken in the NONMEM program on the basis of a chi-square (χ^2^) test of the difference in OFV. A decrease in the OFV of 3.84 units (*P* <0.05) was considered statistically significant. We estimated the area under the concentration-time curve of the first 24 h (AUC_0–24_) using the trapezoidal rule.

### Bootstrap

A nonparametric bootstrap method (n =1,000) was used to study the uncertainty of all PK parameter estimates in the final base model. From the bootstrap empirical posterior distribution, we were able to obtain the 95% confidence interval (2.5 to 97.5% percentile) for the parameters, as described previously [23].

### Covariate screening

The covariates analyzed were age, estimated total body weight, CrCl, SOFA score, APACHE II score, sex, presence of shock, presence of CRRT and the presence of ECMO. Possible covariates were added in a stepwise fashion into the model. Covariates were considered for inclusion in the model if they were biologically plausible and there was improvement in the base model, that is, decrease in objective function (at least 3.84 units), decrease in the unexplained between-subject variability of the parameter, or decrease in residual unexplained variability.

### Dosing simulations

Two sets of Monte Carlo dose simulations were undertaken. We simulated a) different loading doses (15 mg/kg versus 25 mg/kg versus 35 mg/kg) followed by the same CI dose for each loading dose (15 mg/kg/day) in patients on CRRT compared to patients without CRRT; b) different maintenance doses (10 mg/kg/day versus 15 mg/kg/day versus 20 mg/kg/day) in patients on CRRT compared to patients without CRRT after a loading dose of 35 mg/kg. The ability of each dosing regimen to achieve predefined pharmacodynamic targets in both patients, that is, vancomycin concentration of at least 20 mg/L, was also assessed.

### External validation of model

To prospectively evaluate the effectiveness of the model to predict vancomycin concentrations during ECMO therapy, we included all consecutive patients who were treated with ECMO between January and June 2013 and received the same drug regimen (see section, Vancomycin treatment and measurements).

### Statistical analysis

Statistical analyses were performed using the SPSS 13.0 for Windows NT software package (SPSS Inc. 2004). Descriptive statistics were computed for all study variables. The Kolmogorov-Smirnov test was used, and histograms and normal-quantile plots were examined to verify the normality of distribution of continuous variables. Discrete variables were expressed as counts (percentage) and continuous variables as median (25th to 75th percentiles). Demographics and clinical differences between study groups were assessed using the χ^2^ test, Fisher’s exact test, Student’s *t*-test, or Mann-Whitney *U*-test, as appropriate. The coefficient of regression (*r*^2^) was used to indicate the prediction of drug concentrations using the PK model. *P* <0.05 was considered to be statistically significant.

## Results

### Demographics and vancomycin concentrations

We treated a total of 11 patients with vancomycin during ECMO therapy and matched them to another 11 critically ill patients not receiving ECMO (Table 2). ECMO patients had a longer ICU stay (21 (12 to 68) versus 12 (3 to 24) days, respectively) and were more likely to have shock (9/11, 82%, versus 5/11, 45%) than the non-ECMO patients; they also had a higher ICU mortality (6/11, 55%, versus 3/11, 27%). All patients had sepsis on the day of vancomycin initiation and the sources of sepsis were similar in the two groups, mostly pulmonary and abdominal. Blood cultures were positive in almost 30% of patients. All patients were treated concomitantly with other antibiotic therapies. ECMO characteristics remained unchanged over the study period.

**Table 2:** **Characteristics of patients undergoing ECMO and controls**

	**ECMO (n = 11)**	**Controls (n = 11)**
Male, n	4	5
Age, years	43 (19 to 59)	55 (24 to 64)
Estimated weight, kg	70 (46 to 85)	70 (47 to 95)
Estimated body mass index, kg/m^2^	26 (18-29)	24 (18-29)
Medical admission, n	9	8
APACHE II score on ICU admission	22 (3 to 33)	18 (5 to 34)
Mechanical ventilation on ICU admission	9	7
SOFA score on the first day of therapy	11 (5 to 13)	11 (2 to 15)
Time from ICU admission to ECMO, days	3 (0 to 9)	NA
Time from ICU admission to vancomycin therapy, days	7 (4 to 18)	4 (0 to 8) *
Mechanical ventilation on the first day of therapy	11	7
Fluid balance (the day preceding the loading dose), mL/24 h	1959 (−1404 to 5877)	2153 (−1592 to 9686)
Albumin concentration (before the loading dose), g/dL	2.6 (2 to 3.3)	2.8 (2 to 3.8)
Protein concentration (before the loading dose), g/dL	4.2 (2.8 to 4.8)	4.6 (3.4 to 6.4)
Lactate level (before the loading dose), mmol/L	1.1 (0.5 to 17.9)	1.8 (0.7 to 7.9)
CrCl on the first day of therapy, mL/minute (n = 4)	64 (39 to 99)	61 (46 to 109)
CRRT intensity, mL/kg/h (n = 7)	38 (19 to 53)	42 (20 to 50)
Reason for ECMO initiation		
Cardiogenic shock/ARDS/Sepsis, n	5/4/2	2/4/5
VA ECMO/VV ECMO, n	5/6	NA
Overall ICU mortality, n	6	3
**Comorbidities**		
CVD	2	1
COPD/asthma	3	2
Diabetes	3	2
Previous serum creatinine >2.0 mg/dL	1	0
Liver disease	0	1
Solid organ transplant	2	2
Chronic immunosuppressive therapy	3	3
Neutropenia	1	2

Data are presented as count or median (range). *P <0.05. APACHE, acute physiology and chronic health evaluation; SOFA, sequential organ failure assessment; CrCl, creatinine clearance; CRRT, continuous renal replacement therapy; VA ECMO, veno-arterial extracorporeal membrane oxygenation; VV ECMO, veno-venous extracorporeal membrane oxygenation; ARDS, acute respiratory distress syndrome; CVD, cardiovascular disease; COPD, chronic obstructive pulmonary disease; CRF, chronic renal failure; NA, not applicable.

The duration of vancomycin therapy was similar (3 (2 to 9) versus 3 (2 to 10) days) for the two groups. Median vancomycin loading (2,500 (1,610 to 2,975) mg versus 2,450 (1,645 to 3,500) mg) and daily (1,125 (750 to 3,000) versus 1,200 (750 to 2,500) mg) doses were similar for the ECMO and non-ECMO groups. Vancomycin concentrations in ECMO and non-ECMO patients were: 51 (28 to 71) versus 45 (37 to 71) mg/L at T1; 23 (16 to 38) versus 29 (21 to 35) mg/L at T2; 20 (12 to 36) versus 23 (17 to 28) mg/L at T3 (analysis of variance (ANOVA), *P* = 0.53) (Table 3). The percentage of patients with insufficient drug concentrations in the ECMO group was 18% (n = 2/11) at T2 and 36% (n = 4/11) at T3, compared to 0% (n = 0/11) and 9% (n = 1/11), respectively, in the control group.

**Table 3:** **Vancomycin serum concentrations and pharmacokinetics in the ECMO group and in the control group**

	**ECMO (n = 11)**	**Controls (n = 11)**
At T1 (4 h), mg/L	51 (28 to 71)	45 (37 to 71)
At T2 (12 h), mg/L	23 (16 to 38)	29 (21 to 35)
At T3 (24 h), mg/L	20 (12 to 36)	23 (17 to 28)
Vd, L	99.3 (49.1 to 212.3)	92.3 (22.4 to 149.4)
Total CL, L/h	2.4 (1.7 to 4.9)	2.3 (1.8 to 3.6)
AUC_0–24_, mg*h/L	628 (537 to 840)	698 (622 to 753)

ECMO, extracorporeal membrane oxygenation; Vd, drug median volume of distribution; CL, drug clearance; AUC_0–24_, area under the curve of the first 24 hrs of therapy.

### Population pharmacokinetic model building, covariate screening and model evaluation

The time course of vancomycin concentrations was best described by a two-compartment model with exponential residual error and between-subject variability (BSV) on drug clearance (CL), volume of distribution of the central compartment (Vc) and volume of distribution of the peripheral compartment (Vp), but not intercompartmental clearance (Q). Total volume of distribution was expressed as Vd (where Vd = Vc + Vp). This model included zero order input of drug into the central compartment.

The only covariate that statistically improved the base model was the presence of CRRT for CL, which reduced the objective function value by 5.774 (*P* <0.05). To describe this, we created a dichotomous descriptor for the population value for CL, which accounted for the presence of CRRT (CL_CRRT_) or absence of CRRT (CL_NOCRRT_). The presence of ECMO was associated with a 15% increase in CL and 10% decrease in Vc but these relationships did not reach sufficient statistical significance and so were not included in the final covariate model. The final model is represented by:

$$ \mathrm{TVCL}=\mathrm{C}\mathrm{L}*{\mathrm{CL}}_{\mathrm{CRRT}}*{\mathrm{CL}}_{\mathrm{NOCRRT}} $$

where TVCL is the typical value of clearance, CL_CRRT_ is 1 when there is no CRRT present and CL_NOCRRT_ is 1 for patients receiving CRRT.

**Figure 1:**
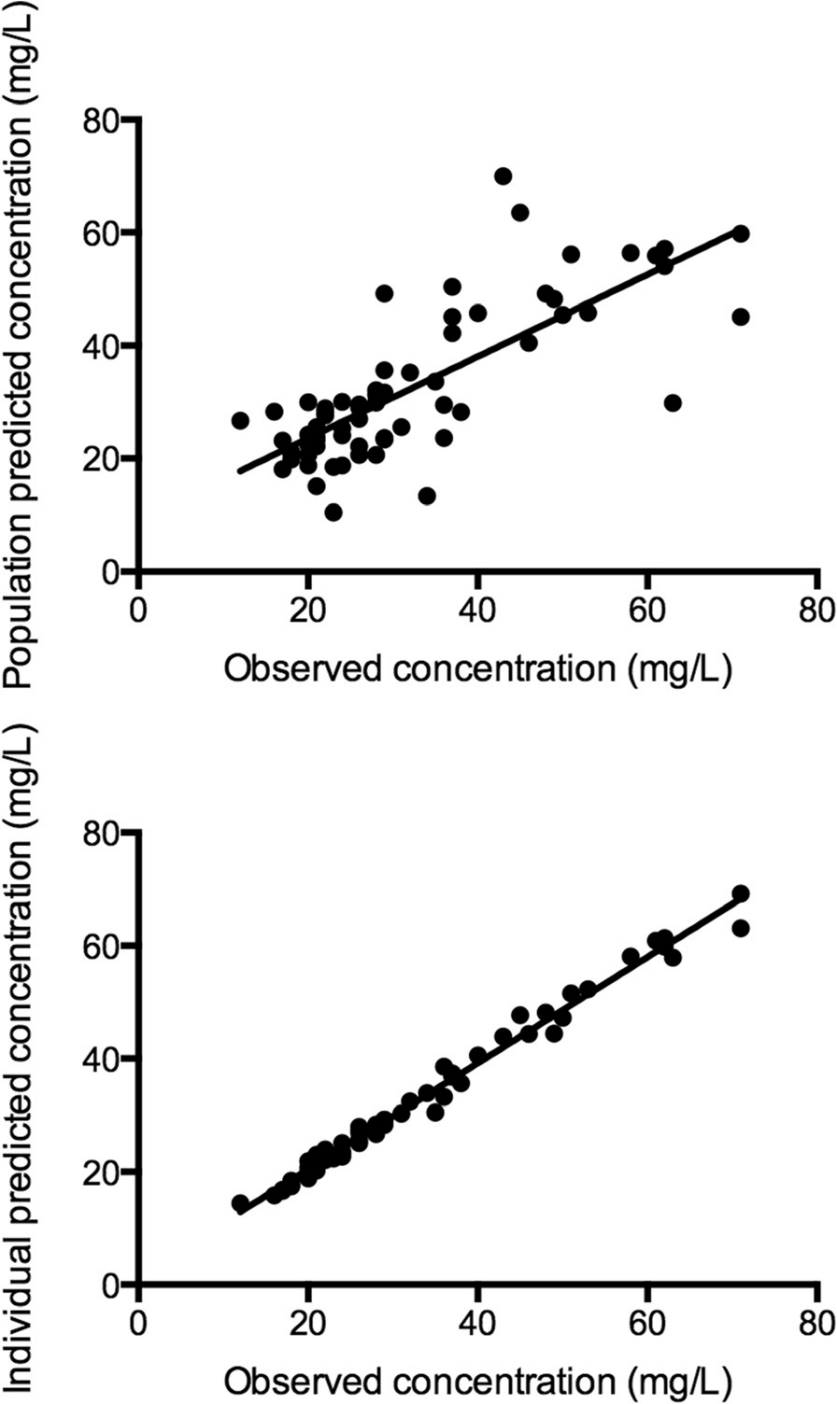
**Goodness-of-fit plots for the final covariate vancomycin pharmacokinetic model.** The top panel presents the population predicted concentrations versus the observed concentrations. The lower panel presents the individual predicted concentrations versus the observed concentrations. For both graphs, the solid line represents the linear correlation (*r* ^2^ = 0.60 population predicted concentrations and *r* ^2^ = 0.99 for the individual predicted concentrations using linear regression).

Goodness-of-fit plots for the final model were evaluated and showed acceptable results in terms of visual or statistical biases (statistically significant systematic deviation away from the observed data) for predicted concentrations (Figure 1). Furthermore, the mean values for all parameters from the bootstrap analysis were similar to those in the final model (Table 4).

**Table 4:** **Bootstrap parameter estimates of the final covariate model**

**Parameter**	**Vancomycin**				
	**Model mean**	**Model standard error %**	**Bootstrap**		
			**Mean**	**95% confidence interval**	
				**2.5%**	**97.5%**
**Fixed effects**					
CL (L/h)	3.7	19.5	3.7	3.1	4.4
CL_CRRT_ (L/h)	0.6	20.2	0.6	0.2	0.8
CL_NOCRRT_ (L/h)	1.0	8.0	0.9	0.6	1.2
Vc (L)	31.8	10.7	31.6	25.5	37.9
Vp (L)	57.1	13.5	68.8	29.9	152.1
Q (L/h)	3.6	36.4	3.7	3.0	4.7
**Random effects BSV, % CV**					
CL (L/h)	16.4	42.0	20.4	0.4	33.9
Vc (L)	47.0	36.2	45.5	31.1	63.7
Vp (L)	101.0	72.8	95.5	34.7	183.1
**Random error**					
RUV, % CV	8.5	35.5	7.7	5.0	10.2

CL, clearance; Vc, volume of distribution of central compartment; CL_NOCRRT_, CL relative to population parameter estimate for CL for patients not receiving continuous renal replacement therapy (CRRT); CL_CRRT_, CL relative to population parameter estimate for CL for patients that were receiving CRRT; Vp, volume of distribution of peripheral compartment; Q, intercompartmental clearance; BSV, between-subject variability; RUV, residual unexplained variability; CV, coefficient of variation.

The final PK parameter estimates for the included patients receiving ECMO versus the control patients are shown in Table 4. The results of the model evaluations confirmed the suitability of this model to describe vancomycin PK in this specific population and for use with dosing simulations.

### Dosing simulations

**Figure 2:**
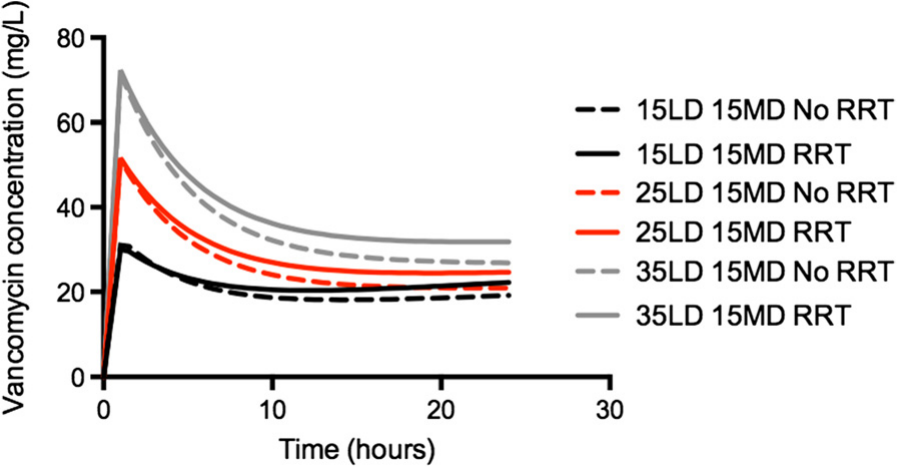
**Effect of different loading doses (LD) on rapid attainment of target vancomycin concentrations (≥20 mg/L).** Daily continuous infusion regimen (MD) was 15 mg/kg/day. The dashed line presents simulations for patients on continuous renal replacement therapy (CRRT) and the solid line those for patients not receiving CRRT.

**Figure 3:**
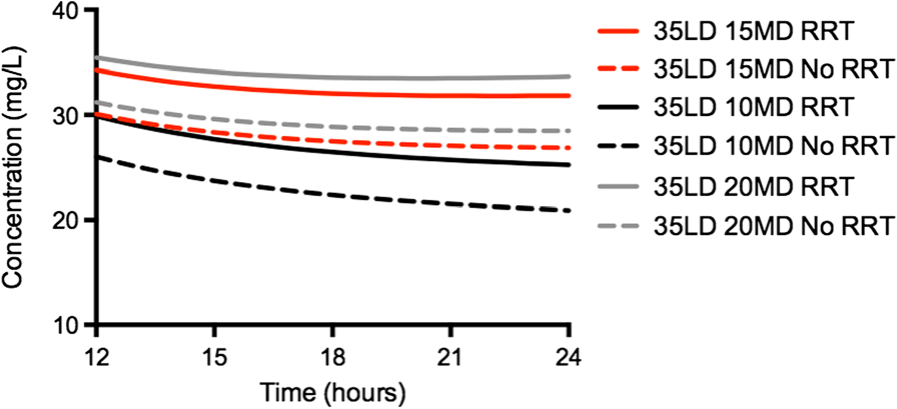
**Effect of different maintenance doses (MD) on rapid attainment of target vancomycin concentrations (≥20 mg/L) after a loading dose of 35 mg/kg.** The dashed line presents simulations for patients on continuous renal replacement therapy (CRRT) and the solid line those for patients not receiving CRRT, who had an estimated creatinine clearance of 100 mL/minute.

Figures 2 and 3 show vancomycin concentrations obtained through simulations for different loading and maintenance doses in the presence/absence of CRRT (50 years of age, 70 kg of weight and a CrCl of 100 mL/minute). Figure 2 shows that even a loading dose of at least 15 mg/kg was necessary to ensure rapid achievement of target vancomycin concentrations. Using the same loading dose in the presence of CRRT did not significantly influence drug concentrations when compared to no use of CRRT. Figure 3 shows that after a 35 mg/kg loading dose, a maintenance CI dose of at least 10 mg/kg/day was required during CRRT, whereas doses of 10 or 15 mg/kg/day were both able to provide drug concentrations between 20 and 30 mg/L in the case of no need for CRRT.

### Model validation

**Figure 4:**
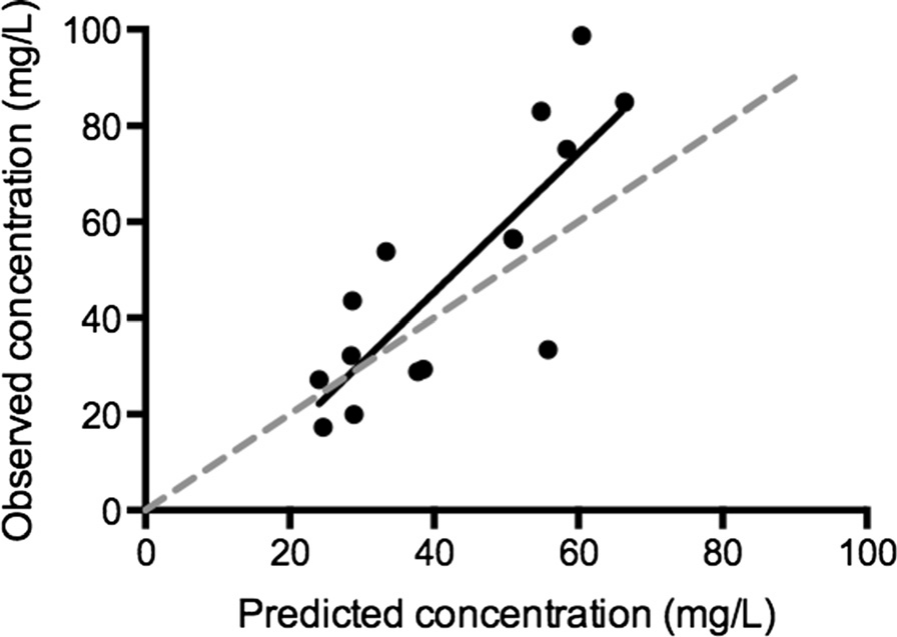
**Observed concentrations from the patients included in the validation cohort versus the concentrations predicted by the model for those patients (linear regression** ***r*** ^**2**^ **0.66;** ***P*** **<0.001).**

Five additional patients (four on VV ECMO and one on VA ECMO) treated with vancomycin during ECMO therapy were identified. Four of the patients were male; the median (IQR) age was 64 (55 to 68) years, weight 71 (70 to 80) kg, and three of the patients were receiving CRRT at an intensity of 21 (21 to 26) mL/kg/h. The patients received a loading dose of 2,485 (2,450 to 2,800) mg of vancomycin and a maintenance continuous infusion of 1,250 (1,050 to 1,750) mg (Table 5). The linear regression analysis confirmed the adequacy of the model by comparing the observed concentrations from the external dataset and those predicted using the model (*r*^2^ of 0.67; *P* <0.001) (Figure 4).

**Table 5:** **Characteristics of the validation cohort of ECMO patients (n = 5)**

**Patient**	**Weight, kg**	**LD, mg**	**DD, mg**	**CrCl, mL/minute**	**CRRT**	**CRRT Intensity, ml/kg**	**ECMO**
1	71	2485	1750	260	N	NA	VV
2	70	2450	1000	53	Y	21	VV
3	80	2800	1250	11	Y	31	VV
4	70	2450	1050	17	Y	21	VV
5	80	2800	3500	267	N	NA	VA

Age (range 45 to 71 years) and gender (4 male/1 female) were not reported to protect the anonymity of the patients. LD, loading dose; DD, daily dose; CrCl, creatinine Clearance; CRRT, continuous renal replacement therapy; VA ECMO, veno-arterial extracorporeal membrane oxygenation; VV ECMO, veno-venous extracorporeal membrane oxygenation; Y, yes; N, no.

## Discussion

This is the first study describing vancomycin PK during continuous infusion of vancomycin in adult critically ill patients undergoing ECMO. Extracorporeal therapy had a non-significant impact on serum vancomycin concentrations when higher doses than the recommended drug regimen were used. Finally, our results highlighted that with this drug regimen, vancomycin concentrations were appropriate in most of ECMO adult patients.

In this cohort, we found similar Vd and CL for vancomycin in patients receiving ECMO when compared to others. These findings are in contrast with neonatal ECMO PK studies in which increased Vd and decreased antibiotic CL were reported [24,25]. Furthermore, it is reasonable to assume that the decreased drug CL observed in neonates was the result of immature hepatic and renal antibiotic metabolic pathways rather than the ECMO circuitry itself [26]. Similarly, the volume of priming fluid for the ECMO system is likely to have a more profound effect on vancomycin Vd in newborns because of a larger priming/blood volume ratio. Thus, data in adult patients may differ significantly from those in neonatal studies, underpinning the need for further PK studies that can be used to optimize antibiotic dosing during ECMO in this setting. Finally, ECMO may have different effects on antibiotic PK depending on the class of antibiotic used. In contrast to studies on aminoglycosides, vancomycin Vd was similar in neonates treated with ECMO compared to controls (0.45 ± 0.18 versus 0.39 ± 0.12 L/kg), whereas half-life was significantly shorter during ECMO (8.3 ± 2.2 versus 6.5 versus 2.0 h, *P* = 0.02) [27].

The Vd of vancomycin was not affected during ECMO therapy but remained substantially higher than values reported for healthy volunteers or non-severely ill patients (that is, from 0.4 to 1.0 L/kg) [10,28]. These findings could be explained in the context of critical illness by the absence of significant circuit drug sequestration, as described in *ex vivo* studies [9]. Moreover, in our study, both fluid balance and serum protein concentrations, which influence the distribution of antibiotics, were similar between groups and did not bias the final observations [28]. In support of our findings, a recently published population PK study of meropenem using similar methodology found no statistically significant difference in meropenem Vd between ECMO and non-ECMO ICU patients (0.45 versus 0.41 L/kg respectively, *P* = 0.21) [29]. A significant increase in Vd was also reported for the antiviral drug, oseltamivir, during ECMO and RRT in adult patients [30]. Nevertheless, the stability at room temperature, protein binding and sequestration in the ECMO circuit of these drugs are different and may explain the discrepancies in Vd that were observed [28]. Moreover, endothelial activation occurring during extracorporeal support, which may promote capillary leakage and increase Vd [31], may differ among patients and with the indication for ECMO therapy, contributing to the large discrepancies among reported data on antibiotic PK in this setting. Importantly, if ECMO causes an increased drug Vd during the initial phase of therapy, then given that the ECMO patients in this study were treated with vancomycin later in their ICU stay, the resulting drug behavior could be associated with a different phase of the inflammatory process, with less capillary leakage and fluid requirement than the non-ECMO patients. Thus, whether our data could have been influenced by a higher than recommended vancomycin dose, to the use of a priming solution volume that is lower than what is used in *in vitro* systems or to a reduced inflammatory status, requires further study.

The increase in vancomycin CL during ECMO therapy was modest, confirming the findings of other studies [30,31]. Moreover, in these patients, total drug CL during CRRT was lower than in patients without CRRT. The lack of increased vancomycin CL during ECMO was probably due to the lack of circuit sequestration, as vancomycin appears to be relatively stable in the circuit [9]. Also, although peripheral VA ECMO is known to cause a significant increase in renal blood flow and as such to promote renal elimination of vancomycin [7], three of the five patients treated with VA ECMO in our cohort had acute kidney injury with ongoing CRRT. It is possible that the use of CrCl or CRRT intensity in the matching process may have underestimated or overestimated the actual drug CL. As such, other biomarkers of renal function, such as cystatin C, have been shown to be more accurate than CrCl to predict vancomycin CL, particularly among patients with normal serum creatinine concentrations [32]. Finally, antibiotic elimination is proportional to the unbound (free) drug concentration; however, total vancomycin concentrations were not predictive of free drug concentrations in ICU patients, suggesting that direct determination of the free component may be desirable in this setting [33]. Further investigations are required to investigate the role of ECMO technique (VA versus VV) and the different biomarkers of renal vancomycin excretion in this setting.

Vancomycin concentrations were comparable in ECMO and non-ECMO groups in the early phase of therapy (24 h), which may be a reflection of the slightly higher loading doses used as well as the continuous administration. However, we cannot draw any conclusions on the effects of ECMO on drug concentrations in the following days of therapy and whether the 15% increase in vancomycin CL would need a more accurate dose adjustment to avoid drug concentrations that will rapidly fall below therapeutic targets remains to be further evaluated. Because intermittent dosing may result in therapeutic failure when dealing with several strains of MRSA [34], continuous infusion has been proposed as an alternative approach to optimize drug concentrations and efficacy [11]. Although not superior to intermittent infusion for clinical effectiveness, continuous infusion may result in a reduced risk of renal toxicity and in a more rapid attainment of therapeutic concentrations [35].

This study has potential limitations. First, the matching process resulted in statistically comparable control and ECMO groups, but it was not possible for us to control for other pathophysiological disturbances that may affect antibiotic PK. Although we accept that the matching process is extremely complicated in critically ill patients, in our opinion the approach we have chosen is sufficient to understand whether ECMO *per se* alters antibiotic PK in adult patients or if the presence of critical illness not requiring ECMO is sufficient to produce such PK abnormalities. This study found that ECMO numerically increased vancomycin clearance by 15%, although this result was not statistically significant. In this setting, the role of CRRT intensity on antibiotic PK needs to be further quantified, as we did not record whether CRRT settings were modified during the first 24 h of therapy. Also, we took into account drug clearance from CRRT intensity but this could be a significant confounder in the interpretation of the results on such a small patient population. Thus, our simulations need to be interpreted with some caution because they were developed from a limited cohort of 11 ECMO patients, with a potentially high degree of heterogeneity. Also, we are aware that a more accurate estimation of the true variability of Vd and clearance would require data from a larger patient population. Nevertheless, we validated the accuracy of the model to predict drug concentrations in this setting, if only in a few patients, and showed that comparison between ECMO and non-ECMO patients including those with CRRT was sufficient. Second, we could not perform a different evaluation for VA and VV ECMO because of the limited cohort, and future models should investigate whether ECMO modality may influence antibiotic concentrations. Third, we did not record data on clinical and microbiological response to vancomycin. Thus, to determine whether optimizing antibiotic concentrations during ECMO leads to improved outcomes requires further study. Fourth, sequestration of drug by the ECMO circuit could not be assessed because we did not perform sampling immediately before and after the ECMO membrane. Fifth, another confounder could be the time elapsed from ECMO initiation to drug administration, as adsorption of vancomycin on the ECMO membrane may vary between new and old membranes/circuits. Finally, the observed high proportion of patients with adequate vancomycin levels during ECMO therapy was related to the higher-than-recommended drug regimen used, while a risk of under-dosing may be significantly present in ECMO patients treated with standard doses.

## Conclusions

In this matched-cohort study, critically ill patients receiving ECMO therapy had similar Vd and vancomycin CL to control non-ECMO patients. Although vancomycin concentrations were quite similar between groups in the early phase of therapy, vancomycin dose adjustment should be considered during CRRT therapy to avoid drug accumulation thereafter. In the first 24 h of treatment, loading and maintenance doses of vancomycin similar to those used in this study appear to be appropriate for patients on ECMO requiring vancomycin therapy.

## Key messages

- Vancomycin concentrations were similar between ECMO and non-ECMO patients in the early phase of therapy
- ECMO treatment was not associated with significant changes in Vd and drug clearance compared with the control patients
- The main determinant of drug CL was the presence of CRRT

## Abbreviations

APACHE: Acute physiology and chronic health evaluation
CL: clearance
CrCl: creatinine clearance
CRRT: continuous renal replacement therapy
ECMO: extracorporeal membrane oxygenation
MRSA: methicillin-resistant *Staphylococcus aureus*
NONMEN: non-linear mixed-effects modeling
OFV: objective function value
PDMS: patient data monitoring system
PK: pharmacokinetic
SOFA: Sequential organ failure assessment
VA: veno-arterial
Vd: volume of distribution
VV: veno-venous
